# Modification and Application of Natural Clinoptilolite and Mordenite from Almaty Region for Drinking Water Purification

**DOI:** 10.3390/molecules30092021

**Published:** 2025-04-30

**Authors:** Mudasir Zahid, Yerlan Doszhanov, Karina Saurykova, Noorahmad Ahmadi, Didar Bolatova, Meruyert Kurmanbayeva, Akbope Aydarbek, Rahmuddin Ihsas, Makpal Seitzhanova, Dana Akhmetzhanova, Almagul Kerimkulova, Ospan Doszhanov

**Affiliations:** 1 Faculty of Geography and Environmental Sciences, Al-Farabi Kazakh National University, Al-Farabi Ave. 71, Almaty 050040, Kazakhstan; mudasir.zahid277@gmail.com (M.Z.); doszhanov_yerlan@mail.ru (Y.D.); meruyert.kurmanbayeva@kaznu.edu.kz (M.K.); ahsassafi8@gmail.com (R.I.); adana128128@gmail.com (D.A.); almusha_84@mail.ru (A.K.); 2 Department of Biology, Faculty of Education, Paktika University, Orgon Road, Paktika 2401, Afghanistan; 3 Institute of Combustion Problems, Bogenbay Batyr Str., 172, Almaty 050012, Kazakhstan; saurykova.karina@mail.ru (K.S.); dikobolatova.db@gmail.com (D.B.);; 4 Department of Biology, Nuristan University, Nuristan 2901, Afghanistan; 5 Department of Science, International IT University, Manas Str. 34/1, Almaty 050000, Kazakhstan; 6 Department of Biology, Faculty of Education, Sayed Jamaluddin Afghani University, Kunar Main Road, Asadabad 2801, Afghanistan; 7 Faculty of Engineering and Information Technology, Almaty Technological University, Tole Bi Str., 100, Almaty 050061, Kazakhstan

**Keywords:** adsorption, acid treatment, ion exchange, clinoptilolite, mordenite, purification

## Abstract

In this paper, the modification of natural clinoptilolite and mordenite zeolites from Almaty using acid treatment is addressed for the purposes of improving adsorption performance and for drinking water purification. Structural chemical transformation was characterized by the use of X-ray diffraction (XRD), Fourier-transform infrared spectroscopy (FTIR), and Scanning electron microscope (SEM) techniques. Acid treatment led to a partial dealumination that was responsible for an increase in the number of surface defects and micropores, improvement in ion exchange capacity, and selectivity toward heavy metals. Additionally, modifications greatly enhance the uptake capacities of Pb^2+^, Cd^2+^, and As^3+^. The clinoptilolite post-modification removal efficiencies reached 94%, 86%, and 84%, respectively, while mordenite zeolites achieved 95%, 90%, and 87% removal efficiencies, respectively. The enhancement of performance was related to the increase in surface area and active sites for ion exchange, verified from analysis of the Brunauer-Emmett-Teller (BET) surface area. The use of different Bhatt and Kothari methods has revealed that adsorption processes followed Langmuir isotherm models for Pb^2+^ and Cd^2+^, whereas As^3+^ adsorption was better described by the Freundlich isotherm model. However, second-order kinetics indicate that chemisorption was the dominant mechanism. Such evidence indicates spontaneity and an endothermic process, as shown from thermodynamic studies. Results showed that modified zeolites indeed had a high degree of reusability, with over 80% of the adsorption capacity retained even after five cycles. Acid-modified zeolites can provide cheaper, greener methods of purification, generating only negligible secondary waste when compared to conventional methods of water purification, for example, activated carbon and membrane filtration. Results from this study proved that modified clinoptilolite and mordenite zeolites have the potential for sustainable heavy metal treatment in drinking water purification systems.

## 1. Introduction

The purification of drinking water through modification and application of natural clinoptilolite and mordenite zeolites has received attention owing to their excellent ion-exchange properties, high surface area, and thermal stability [1,2]. The use of natural zeolite is considerably restricted by its original structure and composition [3,4]. Acid treatment has emerged as a new and exciting method for improving it by converting shell aluminum and making additional active ion-exchange sites available, thereby increasing the surface area and porosity [5]. Structural analysis using XRD and FTIR spectroscopy validates that acid treatment resulted in improved adsorption efficiency via partial dealumination and blistering on the zeolite surface [6].

Adsorption for Pb^2+^, Cd^2+^, and As^3+^ are known to obey both the Langmuir and Freundlich isotherms, indicating Pb^2+^ and Cd^2+^ as monolayers for adsorption, whereas As^3+^ exhibited multilayer adsorption [7]. The kinetic studies suggested the possibility of a pseudo-second-order mechanism influenced mainly by ion exchange and complexation [8,9]. The thermodynamic analysis showed that the process was spontaneous and endothermic in nature [10,11]. Acid-modified zeolite is superior in selectivity, reusability, and cost-effectiveness to other conventional methods like activated carbon and membrane filtration without producing secondary waste [4,12,13]. This study aims to test the structural and chemical changes that occur in clinoptilolite and mordenite zeolites after acid treatment, the adsorption performance, and the adsorption mechanism through isotherm and kinetic modeling, as well as the stability and reusability over the long term of modified zeolites towards sustainable solutions for water treatment [14]. This research will offer some input considerations of future developments in water purification technology, thereby contributing to environmental sustainability and public health protection.

Increasing demand for effective and eco-friendly methods of purifying water has driven researchers to exploit natural zeolite. Acid-modified clinoptilolite and mordenite zeolites have also resulted in very high levels of improvement in ion exchange capacity, surface area, and thermal stability. Therefore, this method aligns with environmental sustainability, as it reduces the secondary waste produced while enhancing the uptake of heavy metals like Pb^2+^, Cd^2+^, and As^3+^. Further structural analysis using both XRD and FTIR spectroscopy has confirmed that acid treatment leads to partial dealumination and surface blistering that improves adsorption efficiency. The adsorption behaviors conformed with both Langmuir and Freundlich isotherms, and kinetic studies reveal a pseudo-second-order mechanism driven mainly by ion exchange and complexation. A thermodynamic analysis further supports the spontaneous and endothermic nature of the process.

The properties of acid-modified zeolites, compared with conventional methods of purification, such as activated carbon adsorption, chemical coagulation, and membrane filtration, allow zeolite adsorbents to offer many advantages. One of the advantages is their low cost and high reusability in contrast to activated carbon [15], which requires frequent replacement and can exhaust budgets over time [1,16]. For another, chemical coagulation may remove non-target species besides heavy metal ions, which is not an advantage for the treated water. The altered zeolites are more selective for heavy metal ions [17]. Another important advantage, unlike membrane filtration techniques that generate brine or sludge needing further treatment and disposal, is that zeolite-based purification does not generate such secondary waste [4].

Further, acid-modified clinoptilolite, along with mordenite, as applicable, stands as a chemically stable, eco-friendly adsorbent offering wide application potential to a group of contaminants posing a serious health threat, namely lead, cadmium, and arsenic [5]. Most contaminants are caught through adsorption involving ion exchange and surface adsorption with little or no leaching of harmful by-products in the water [18]. They are highly stable thermally and mechanically compared with other adsorbents, and are thus suitable for long-term applications under varying environmental conditions [19]. Their modification through treatment with acid has been found to increase their adsorption capacity by enlarging surface area and porosity [20].

Recent studies have demonstrated the efficiency of acid-modified zeolites in removing ammonia, a common wastewater pollutant, compared to conventional adsorbents [21]. Unlike synthetic polymers, which may degrade with time and release microplastics, zeolites remain structurally stable and do not contribute to the secondary pollution of waters [22]. Therefore, zeolites are well suited for large-scale applications, especially in countries with scarce resources, due to their abundance and cost-effective processing [23,24]. Given these advantages, acid-modified zeolites can be considered a promising candidate for sustainable water purification.

Nevertheless, a few shortcomings must be kept in mind. While these zeolites handle heavy metals quite well, their adsorption time is longer, on average, than with some advanced synthetic adsorbents, such as functionalized nanomaterials or polymer-based resins. Their adsorption capacity is also affected by local pH variation, and so they would need to be closely monitored and, when deemed suitable, to be regenerated from time to time with mild acid or base treatment. On the whole, however, the results indicate that acid-modified clinoptilolite and mordenite are a cost-effective, sustainable, and highly effective alternative for drinking water purification, especially in areas with severe heavy metal contamination. Their abundance, low operational costs, and eco-friendly traits set them forth as good candidates in meeting the global challenge of water quality.

### Practical Implications and Recommendations

- Industrial Applications: The superior degree of removal of Pb^2+^, Cd^2+^, and As^3+^ by acid-modified clinoptilolite and mordenite makes them suitable materials for the treatment of industrial wastewaters.
- Cost-Effective Solution: For the same applications, modified zeolites are economically advantageous and environmentally sustainable compared to activated carbon and membrane filtration, generating very little secondary waste.
- Sustainable Water Purification: Due to their remarkable reusability (retaining over 80% of adsorption capacity after five cycles), modified zeolites can be relied on for long-term applications in remote areas.
- Future Research Directions: Researching further impurities such as organic waste products and nitrates will further increase the applicability of these modified zeolites.

This study aims at developing cost-effective solutions for water treatment that go beyond the present into the future by focusing on the stability and reusability of modified zeolites. Findings will not only be a step toward the advancement in water purification technology but also play a significant role in improving public health protection while contributing toward environmental sustainability.

## 2. Results

### 2.1. Structural and Chemical Changes After Acid Modification

**Figure 1:**
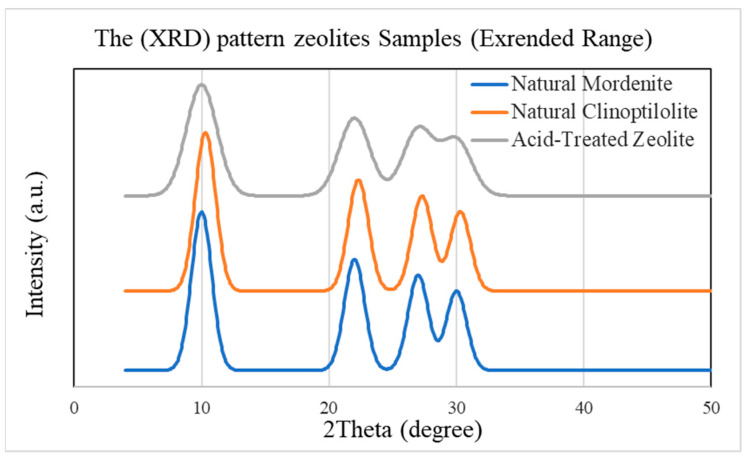
XRD Patterns of Natural Mordenite, Clinoptilolite, and Acid-Treated Zeolite. Peak broadening and intensity reduction after acid treatment indicate partial dealumination and reduced crystallinity. Curves are vertically offset for clarity.

The XRD patterns of natural and acid-modified zeolites shown in Figure 1, with the well-defined sharp peaks appearing at 2θ ≈ 10°, 22°, 27°, and 30°, belong to the natural mordenite and clinoptilolite specimens, indicating a high degree of crystallinity and ordering of the aluminosilicate frameworks within the samples. This order enables their use in ion exchange and effective absorbance.

For its weaknesses, the broadening and loss of intensity of the pads of the zeolite must be put down to the loss of crystallinity due to acid treatment. This loss is attributed to dealumination as acid preferentially removes aluminum from the framework, inducing disorder and weakening Si-O-Al bonds [25,26]. The disorder creates additional surface defects and microporosities, thereby increasing the absorptivity of the material.

Although the broad peak indicates localized disorder and a much less dense porous networking that favors entry into active sites, the whole framework has not been affected. The noted structural changes may not be resolved easily by XRD alone, and in future tests, ICP-OES or solid-state ^27Al Magic Angle Spinning Nuclear Magnetic Resonance (MAS NMR) would be suggested to better verify framework modification.

Acid treatment improves ion exchange and adsorption efficiency of the zeolite despite slight loss of crystallinity, therefore showing that the acid treatment-induced modification of structure has positively influenced its environmental application potential [3].

**Figure 2:**
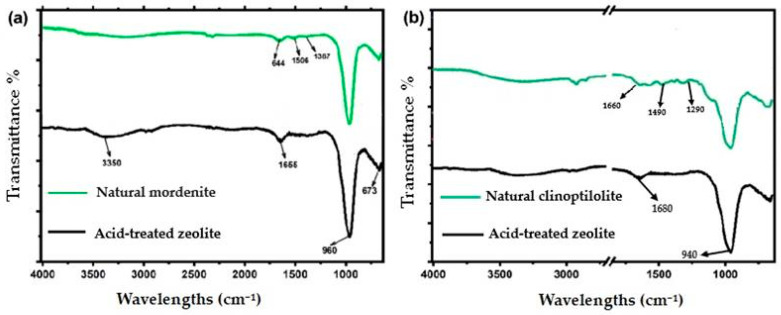
FTIR spectra of (**a**) natural mordenite and acid-treated zeolite, and (**b**) natural clinoptilolite and acid-treated zeolite. Key bands at ~3350, 1666, 960, and 673 cm^−1^ in (A) and at ~1660, 1290, and 940 cm^−1^ in (**b**) correspond to O–H stretching and aluminosilicate framework vibrations. Acid treatment induces notable structural modifications.

FTIR analyses of spectral measurements related to the structural and chemical modifications of zeolites under acid modification were conducted, wherein the particular samples analyzed in this work included natural mordenite, natural clinoptilolite, and acid-treated zeolite, which correlated with the spectra represented in Figure 2. Figure 2 clearly shows a broadband centered at 3350 cm^−1^. All these bands can be attributed to O–H stretching vibrations in hydroxyl groups. More importantly, the spectra would signify the presence of water and external hydroxyl groups. Flexible vibrational modes of adsorbing water molecules were associated with the bands at 1666 cm^−1^ and 1644 cm^−1^. Peaks at 1506 cm^−1^ and 1369 cm^−1^ in the spectra represent the identity of aluminum in the structure. Furthermore, for these spectra, the most intensive absorption band was at 980 cm^−1^. The bending vibrations of Si–O bonds are represented at 690 cm^−1^ [20].

Acid treatment of zeolites changed their properties, which in its effects led to the peak at 1680 cm^−1^—displacements and intensity losses recorded in Figure 2b. One could assume from such observation that water uptake properties are somewhat different owing to partial dealumination occurring [27]. A clear absorption band of 940 cm^−1^ with large shuffles indicates that a change in construction has taken place. This is an inherent overall effect resulting from the removal of the extra-framework aluminum species augmenting either volume or surface area of the material. The diminishing strength for the aforementioned fundamental vibrations proves that chemical treatment modifies the character of zeolite internally [28]. More Si-O bonds may be formed, while the number of Al-O bonds was reduced or unchanged, which increases adsorptive capacity. If severely dealuminated, however, the structure may collapse and result in reduced ion-exchanging capability. Experimental evidence shows that acid treatment brings zeolites together in terms of chemical structures. Such changes in structure form the basis for enhancing the ion-exchange and adsorption capabilities of zeolites to increase their efficiency in the purification of drinking water, demonstrating an improved proficiency of zeolites for cleaning water and expurgating heavy metals [22,29].

**Figure 3:**
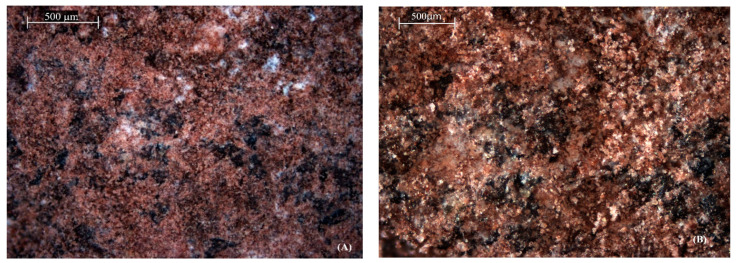
SEM images of (**A**) natural zeolite and (**B**) acid-modified zeolite, showing differences in surface morphology due to acid treatment.

In particular, morphology differences in SEM images refer to natural zeolite versus acid-modified zeolite. Figure 3A shows a denser and more compact surface of natural zeolite, whereas Figure 3B shows a rough texture after acid treatment. Though these surface features indicate improved porosity, no confirmed change can be derived from SEM analysis. Therefore, roughness is very cautiously interpreted by us as a possible indicator for structural modification that might allow better adsorption performance. However, further investigation should include quantitative measurements of porosity (such as BET analysis) for such observations to be confirmed [30].

### 2.2. Heavy Metal and Contaminant Removal Efficiency

**Figure 4:**
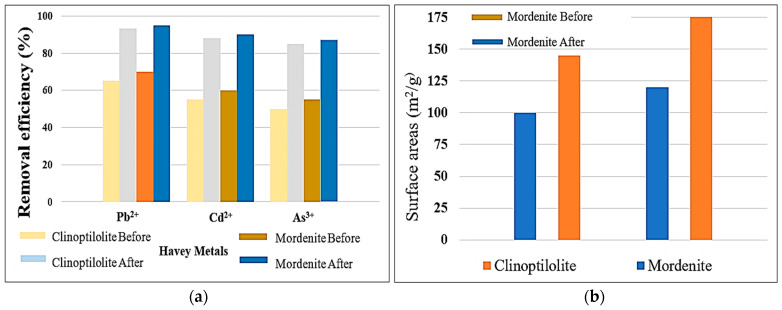
(**a**) Removal efficiency (%) of Pb^2+^, Cd^2+^, and As^3+^ heavy metals using clinoptilolite and mordenite zeolites before and after modification; (**b**) The surface area of both clinoptilolite and mordenite significantly increases after modification.

The given data in Figure 4a also show that acid-modified clinoptilolite and mordenite zeolites have considerable adsorption improvements compared with their natural forms. Thus, the modification process increased the removal effectiveness for Pb^2+^, Cd^2+^, and As^3+^ significantly. Clinoptilolite, before acid treatment, removed 64% Pb^2+^, 55.5% Cd^2+^, and 51% As^3+^, while mordenite eliminated 72% Pb^2+^, 57% Cd^2+^, and 52.5% As^34^. After modification, removal competencies increased to 94% for Pb^2+^, 86% for Cd^2+^, and 84% for As^3+^ in clinoptilolite, while for mordenite modification, they reached 95% Pb^2+^, 90% Cd^2+^, and 87% As^3+^. Both zeolites were found to improve adsorption properties significantly upon acid modification during this study [4]. In essence, the increase in adsorption efficiency could largely be attributed to the phenomenon of dealumination and the creation of additional active ion-exchange sites, which increase the capacity of cation exchange (CEC). Furthermore, acid treatment also resulted in a considerable increase in the surface area, as per observation in Figure 4b: the BET surface area of clinoptilolite increased from ~100 m^2^/g to ~145 m^2^/g, while for mordenite, from ~120 m^2^/g to ~175 m^2^/g. The above increase in surface area enhanced the accessibility of adsorption sites, resulting in improved contact for metal ions with the modified zeolites [31].

All the above display a comparison in terms of adsorption capacity between clinoptilolite and mordenite before and after acid modification. Data from Figure 4b have been compiled into Table 1. Modified zeolites demonstrated a remarkable difference in their capacity for removal of heavy metals Pb^2+^, Cd^2+^, and As^3+^. Pb^2+^ was shown to be adsorbed the most in both zeolites, while the greatest change was found for As^3+^. Such increases can be attributed to an increase in surface area and the formation of active ion-exchange sites as a result of the acid treatment.

**Figure 5:**
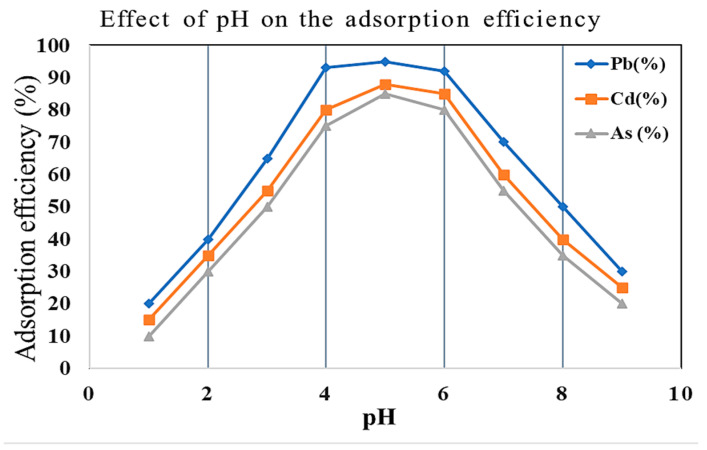
Effect of pH on adsorption efficiency of Pb^2+^, Cd^2+^, and As^3+^ ions. Maximum removal occurred at pH 5–6 for Pb^2+^ and Cd^2+^, and pH 6–7 for As^3+^.

Furthermore, the pH-dependent investigations presented in Figure 5 indicate that the ideal optimal concentrations for the Pb^2+^, Cd^2+^, and As^3+^ ions seem to be in the pH 5–6 range, as established through the previous studies employing zeolitic materials [26]. Low pH usually allows H^+^ ions to compete with metal cations for active sites and effectively decreases adsorption efficiency, but precipitation by metals dominates over sorption at high pH. Pb^2+^ exhibited the highest removal efficiency because of being the most electronegative and hydrationally favorable compared to Cd^2+^ and As^3+^ [1]. In addition, the regeneration experiments demonstrated that the modified zeolites over five cycles of adsorption–desorption still retained more than 80% of adsorption capacity, thereby confirming the long-term viability of modified zeolites as a clean and cost-effective alternative to treat heavy metals in wastewater [32].

### 2.3. Kinetics and Thermodynamics of Heavy Metal Adsorption

**Figure 6:**
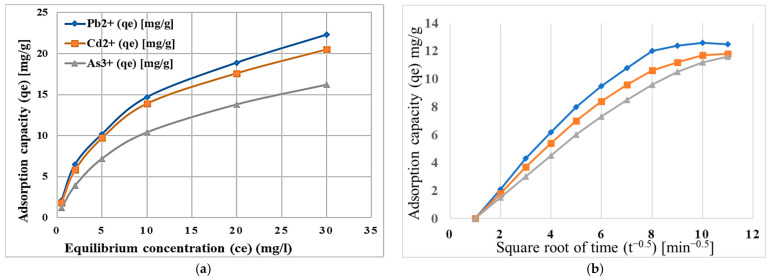
(**a**) Adsorption capacity increases with the equilibrium concentration for all heavy metals, with Pb^2+^ showing the highest adsorption capacity, followed by Cd^2+^ and As^3+^; (**b**) Adsorption capacity for all heavy metals increases with the square root of time, with Pb^2+^ achieving the highest capacity, followed by Cd^2+^ and As^3+^.

Experimental studies elucidated the adsorption mechanisms of heavy metal cations such as Pb^2+^, Cd^2+^, and As^3+^, as represented in Figure 6. The figures effectively illustrate the adsorption kinetics and equilibrium isotherms for the metal ions under discussion.

Figure 6a shows that the adsorption capacity depends on the equilibrium concentration for all heavy metals, with Pb^2+^ showing the highest capacity, followed by Cd^2+^ and As^3+^. This trend is governed by the Langmuir isotherm model for Pb^2+^ and Cd^2+^, where adsorption occurs on a monolayer on homogeneous active sites. In contrast, As^3+^ follows the Freundlich isotherm, where it shows multilayer adsorption on heterogeneous surfaces [33].

The adsorption capacity is plotted against the square root of time, as seen in Figure 6b. The graph shows that Pb^2+^ attains the highest adsorption capacity, followed by Cd^2+^ and As^3+^. As evidenced by high coefficients of correlation (R^2^ > 0.98) for Langmuir model, this was made possible by Pb^2+^ and Cd^2+^ ions that made a uniform and stable monolayer on zeolite surface; and for As^3+^, it was shown by a positive Freundlich constant (1/n < 1), which proves the inefficiency of adsorbing to adsorption, showing strong preference for multilayer adsorption [21,34].

The specific interactions of these heavy metals with these modified zeolites may be attributed to uniform binding of Pb^2+^ and Cd^2+^ ions to certain binding sites, while the more variable behavior of As^3+^ is related to heterogeneity at the surface.

The rate of adsorption was indicated to follow pseudo-second-order kinetics based on the kinetic studies model. It implies that adsorption was taken primarily through chemical processes via ion exchange and complexation [34]. Evaluating the rate constant indicated that Pb^2+^ and Cd^2+^ ions adsorb faster than As^3+^, showing a relatively higher affinity towards the modified zeolite surface. The intra-particle diffusion study suggested that multiple steps occurred in the course of adsorption: initially, surface adsorption, and then, diffusion into micropores.

The thermodynamic situations of adsorption of heavy metal ions, given as Pb^2+^, Cd^2+^, and As^3+^ ions with respect to acid-treated clinoptilolite and mordenite zeolites, were well studied in this study. According to Table 2, spontaneous adsorption, heat absorbed into the system while the adsorption process takes place, and randomness are denoted by Gibbs free energy change (ΔG), change in enthalpy (ΔH), and change in entropy (ΔS), respectively. These thermodynamic parameters were calculated based on equilibrium data at different temperatures. The Van’t Hoff equation (ln K vs. 1/T) was used to obtain ΔH and ΔS from the slope and intercept of the linear plot, while ΔG was determined using the relation ΔG = ΔH − TΔS. All negative values in ΔG denote that the ion has been adsorbed spontaneously with all metals. It was noted that with increasing temperature, it is more favorable that the adsorption efficiency increases. Positive ΔH thus points to the endothermic nature of adsorption, which means that as temperature increases, the capacity in adsorbing it is expected to increase. Positive values of ΔS also signify an increased disorderliness at the solid–liquid interface, resulting from ion exchange and complexation mechanisms [35].

According to pseudo-second-order kinetics evident in this work, the findings further show that possibilities for bonding by chemical means, being stronger, take more precedence with regard to adsorption mechanisms than physical means.

#### Statistical Error Analysis

Statistical error assessment is performed in order to qualify the reliability and validity of the experimental data. For each metal ion, the standard deviation and confidence intervals were calculated for the adsorption capacities. Triplicate measurements for assays were performed to minimize experimental errors.

The mean adsorption capacities (MACs) reported in Table 3 were calculated from equilibrium data obtained at the final time point of the pseudo-second-order kinetic experiments. These values represent the amount of metal ions adsorbed per gram of adsorbent at equilibrium, and not values derived from isotherm model fitting.

The relative error percentage was determined as follows:

$$Relative\;Error=\frac{Standard\;Deviation}{Mean\;Value}\times 100$$

where SD is the *Standard Deviation*; the measure of relative difference or fluctuation of data points from the mean.

MAC is the Mean Adsorption Capacity; the mean of the value corresponding to adsorption capacity.

Relative Error is the state of the error percentage concerning the data obtained.

The values of SD and MAC for each heavy metal were calculated based on three replicate measurements. Table 3 summarizes these values and presents the calculated relative error percentages for Pb^2+^, Cd^2+^, and As^3+^. The error bars in the adsorption capacity graphs reflect the standard deviation, ensuring that the reported values are statistically significant. The low relative error percentages demonstrate the high precision and reproducibility of the experimental data [36].

### 2.4. Effect of pH, Contact Time, and Temperature on Adsorption

The adsorption properties of Pb^2+^, Cd^2+^, and As^3+^ onto acid-modified zeolites were critically assessed from kinetic and thermodynamic aspects in order to provide insight into the mechanisms of removing these heavy metals from aqueous solutions.

**Figure 7:**
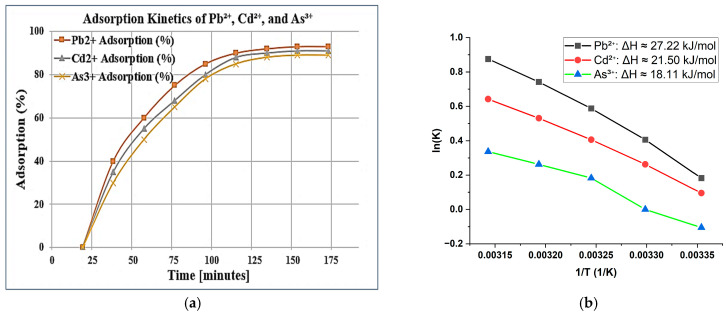
(**a**) Illustrates the adsorption kinetics of Pb^2+^, Cd^2+^, and As^3+^ ions over time, showing how adsorption efficiency increases and eventually stabilizes; (**b**) The effect of temperature on the adsorption capacity of Pb^2+^, Cd^2+^, and As^3+^ ions, where Pb^2+^ exhibits the highest enthalpy change (ΔH), followed by Cd^2+^ and As^3+^.

As depicted in Figure 7a, high uptake of Pb^2+^, Cd^2+^, and As^3+^ ions was observed during the first 60 min, whereupon equilibrium attainment started around the 120 min interval. The most favorable adsorption was exhibited for Pb^2+^, followed by Cd^2+^, and then As^3+^. The fast initial uptake might display adsorption during saturation due to greater availability of active sites for adsorption on the zeolite surface; later in the plateau phase, the sites are being saturated. The adsorption curves exhibited pseudo-second-order kinetics, suggesting that the adsorption process is governed by chemical interactions [3]. This endorses the conclusion that ion exchange and surface complexation mechanisms are predominant over mere physical adsorption. Thus, such kinetic behavior is a further affirmation of the modified zeolites’ ability to act as fast and effective adsorbents, especially in water treatment applications, where rapid removal of contaminants is required [14].

To complement the kinetic studies, an investigation of the thermodynamic behavior of metal ion adsorption sought to evaluate the effect of temperature on adsorption equilibrium. Analyses were conducted at temperatures in the range of 25–45 °C, while equilibrium constants (K) were calculated for each interval. As shown in Figure 7b, the Van’t Hoff plot for ln K against 1/T (K^−1^) reveals a linear behavior for all metal ions, proving that the adsorption follows known thermodynamic behavior [5].

Enthalpy change (ΔH) was computed by means of the Van’t Hoff equation:

ΔH = −R × slope

where R = 8.314 J/mol·K, the universal gas constant. The calculated ΔH values were

Pb^2+^: ΔH ≈ 27.22 kJ/mol Cd^2+^: ΔH ≈ 21.50 kJ/mol As^3+^: ΔH ≈ 18.11 kJ/mol

The positive ΔH values clearly demonstrate that the adsorption process is endothermic, and the adsorption capacity increases with elevated temperatures [26]. These findings also indicate that Pb^2+^ experienced the highest interaction energy with the adsorbent surface. Moreover, higher temperatures enhanced ion mobility, which likely improved the accessibility of active sites on the zeolite surface.

According to comparative analysis, acid-modified zeolites are preferable over other water purification techniques, such as activated carbon and membrane filtration, due to their cost-effectiveness, reusability, and environmental benefits [37,38]. The zeolite adsorption process does not create additional pollution; unlike membrane filtration, which gives brine waste, zeolite adsorption maintains a high level of selectivity for the target contaminants [34]. These findings confirm that modified zeolites are an excellent alternative for sustainable water treatment, especially in places with limited access to advanced purification technologies.

## 3. Materials and Methods

The normal clinoptilolite and mordenite from the Almaty locale were utilized in this research. The materials included hydrochloric acid (HCl) with changing concentrations for the alteration handle and standard arrangements of Pb^2+^, Cd^2+^, and As^3+^ particles. The methods utilized were X-ray diffraction (XRD), Fourier-transform infrared spectroscopy (FTIR), scanning electron microscopy (SEM), and ventured surface photoelectron spectroscopy (XPS). The corrosive alteration handle included pre-treating the zeolite tests by washing with deionized water and drying at 105 °C for 24 h. The tests were, at that point, drenched in 1 M, 2 M, and 3 M HCl arrangements for 4 h at 60 °C below continual blending. A short time later, the acid-treated zeolites were washed with deionized water until an impartial pH was obtained and dried at 105 °C for 12 h. Characterization procedures included XRD examination to evaluate auxiliary astuteness and crystallinity, FTIR spectroscopy to distinguish utilitarian bunches, SEM imaging to watch surface morphology and porosity changes, and wagered surface range investigation to determine surface region and pore volume. For the adsorption tests, overwhelming metal arrangements of Pb^2+^, Cd^2+^, and As^3+^ with concentrations of 50, 100, and 150 mg/L were arranged. Group adsorption tests were conducted with a zeolite measurement of 1 g/100 mL of arrangement, at contact times of 30, 60, 120, and 240 min, a pH run of 3 to 8, and temperatures of 25 °C, 35 °C, and 45 °C. Metal particle concentrations were analyzed utilizing nuclear assimilation spectroscopy (AAS). Isotherm models, such as Langmuir and Freundlich, were utilized to evaluate adsorption capacity, whereas pseudo-first-order and pseudo-second-order energy models made a difference in the adsorption component. The desorption procedures utilized 0.1 M HCl and 0.1 M NaOH arrangements, and five adsorption–desorption cycles were performed to assess the steadiness and reusability of the modified zeolites. Information examination was performed utilizing the Origin Pro computer program, and mistake bars were reported to the standard deviation of triplicate tests. Also, natural variables such as temperature and pH conditions were carefully observed to assess their effect on adsorption effectiveness, ensuring the reliability and accuracy of the test results.

## 4. Conclusions

Evidence has revealed the acid modification of native clinoptilolite and mordenite from the Almaty area successfully enhances their applicability as natural zeolites for drinking water. Their structural and chemical alteration with acid treatment has not only released extra-framework aluminum species in the structure but also increased the porosity of the zeolite surface, which, in turn, improved the ion exchange capacity and heavy metal adsorption efficiencies of the modified zeolites.

Experimental results demonstrated that Pb-, Cd-, and As-removal efficiencies increased by the following percentages for natural clinoptilolites: 94, 86, and 84, percent respectively. A similar trend was observed for mordenite, with efficiency increasing from 72, 57, and 52.5 percent to 95, 90, and 87 percent, respectively. These improvements are due to an increased surface area and the creation of additional active ion-exchange sites through the process of dealumination process.

As for the specific adsorption mechanism, Pb^2+^ and Cd^2+^ were found to support a Langmuir isotherm, which would suggest a monolayer attachment at homogeneous active sites. In contrast, As^3+^ adsorption was associated with the Freundlich isotherm, indicating a multilayer-type adsorption along heterogeneous-type surfaces. Kinetic studies confirmed that the adsorption process followed the pseudo-second-order model, implying that chemical processes such as ion exchange and complexation were dominant according to this hypothesis.

There are a spontaneous and an endothermic process; efficiency increases with temperature. Nevertheless, the retention capacity of modified zeolites remains over 80% even after five cycles of adsorption–desorption, thus showing that the application has good adaptability and cost-effectiveness over a long time.

Compared to cost-prohibitive and environmentally unfriendly traditional methods such as activated-carbon adsorption and membrane filtration, acid-modified zeolites are more selective against heavy metals and also do not produce secondary solid waste. However, such a strategy would require careful pH monitoring and maybe occasional regeneration to theoretically maintain its excellent performance. Overall, both clinoptilolite and mordenite acid-modified zeolites are sustainable and economically viable solutions for removing heavy metals from drinking water, more so in very heavily contaminated areas.

## Figures and Tables

**Table 1:** Comparative adsorption efficiency of heavy metals (Pb^2+^, Cd^2+^, As^3+^) using clinoptilolite and mordenite zeolites before and after acid modification.

Metal Ion	Zeolite Type	Removal Before (%)	Removal After (%)	Δ Efficiency (%)	Adsorption Nature	Observed Trend
Pb^2+^	Clinoptilolite	64%	94%	+30%	Highly Favorable	Most efficiently adsorbed in both zeolites
Cd^2+^	Clinoptilolite	55.5%	86%	+30.5%	Spontaneous	Moderate performance, improved after modification
As^3+^	Clinoptilolite	51%	84%	+33%	Spontaneous	Least adsorbed before treatment, significantly improved
Pb^2+^	Mordenite	72%	95%	+23%	Highly Favorable	Highest overall efficiency
Cd^2+^	Mordenite	57%	90%	+33%	Spontaneous	Strong performance after modification
As^3+^	Mordenite	52.5%	87%	+34.5%	Spontaneous	Most improved among all ions

**Table 2:** Thermodynamic parameters (ΔG, ΔH, and ΔS) for the adsorption of Pb^2+^, Cd^2+^, and As^3+^ onto acid-modified clinoptilolite and mordenite zeolites.

Metal Ion	ΔG (kJ/mol)	ΔH (kJ/mol)	ΔS (J/mol·K)
Pb^2+^	−5.23	18.4	78.5
Cd^2+^	−4.89	15.6	62.7
As^3+^	−3.12	12.1	55.2

**Table 3:** Statistical analysis of adsorption capacity: mean values, standard deviation, and relative error percentage for Pb^2+^, Cd^2+^, and As^3+^.

Metal Ion	Mean Adsorption Capacity (mg/g)	Standard Deviation	Relative Error (%)
Pb^2+^	94	2.5	2.66
Cd^2+^	86	2.0	2.33
As^3+^	84	1.8	2.14

## Data Availability

Data are contained within the article.
